# Blood ammonia and eye-hand coordination negatively affect health-related quality of life in women with minimal hepatic encephalopathy

**DOI:** 10.1007/s11136-025-03920-3

**Published:** 2025-04-01

**Authors:** Daniela Batallas, Juan José Gallego, Franc Casanova-Ferrer, Alessandra Fiorillo, Pablo Rivas-Diaz, Adrià López-Gramaje, Yaiza M. Arenas, Luis Aparicio, Desamparados Escudero-García, Lucía Durbán, María-Pilar Rios, Salvador Benlloch, Amparo Urios, Vanesa Hidalgo, Carmina Montoliu, Alicia Salvador

**Affiliations:** 1 Laboratory of Social Cognitive Neuroscience, Department of Psychobiology and IDOCAL, University of Valencia, 46010 Valencia, Spain; 2 INCLIVA Biomedical Research Institute, 46010 Valencia, Spain; 3 Department of Pathology, Faculty of Medicine, University of Valencia, 46010 Valencia, Spain; 4 Anatomy and Embryology Department, University of Valencia, 46010 Valencia, Spain; 5 Servicio de Medicina Digestiva, Departamento de Medicina, Hospital Clínico Universitario de Valencia, Universidad de Valencia, 46010 Valencia, Spain; 6 Servicio de Medicina Digestiva, Hospital Arnau de Vilanova, 46015 Valencia, Spain; 7 Universidad Cardenal Herrera-CEU Universities, 46115 Valencia, Spain; 8 CIBERehd, Instituto de Salud Carlos III, 28029 Madrid, Spain; 9 Department of Psychology and Sociology, Area of Psychobiology, University of Zaragoza, Teruel, Spain; 10 Spanish National Network for Research in Mental Health CIBERSAM, 28029 Madrid, Spain

**Keywords:** Minimal hepatic encephalopathy, Blood ammonia, Eye-hand coordination, Quality of life, Sex difference

## Abstract

**Purpose:**

Minimal hepatic encephalopathy (MHE) is common in cirrhosis, leading to cognitive impairment and eye-hand coordination (EHC) alterations. Hyperammonemia plays a key role in MHE, contributing to motor and cognitive deficits. Elevated blood ammonia levels and impaired EHC correlate with neuropsychiatric dysfunction, yet their direct impact on health-related quality of life (HRQoL) is complex. This study examines the associations between blood ammonia, EHC, and HRQoL, and the moderating influence of sex on these associations.

**Methods:**

Eighty-seven cirrhotic patients (67 male) and 23 healthy volunteers (11 male), aged 44–80 years, performed the Psychometric Hepatic Encephalopathy Score (PHES) for MHE diagnosis, the Vienna Test System, bimanual and visuomotor tests, and completed the SF-36 questionnaire to measure HRQoL. Blood samples were taken to test ammonia levels.

**Results:**

Results indicated a significant association between elevated blood ammonia and impaired EHC among cirrhotic patients. However, increased blood ammonia and EHC did not directly predict HRQoL. Moderated moderation analysis revealed that women with MHE showed greater sensitivity to hyperammonemia and EHC deficits in tasks requiring fine motor control and stability skills (aiming, tapping, and bimanual coordination), which were linked to lower HRQoL in both physical and mental domains. In women without MHE, alterations in linear tracking were linked to worse HRQoL. These effects were not observed in men.

**Conclusions:**

The findings underscore the sex-specific impacts of MHE, with women disproportionately affected by ammonia-related motor impairments and their subsequent influence on HRQoL. These results could contribute to developing targeted strategies to improve outcomes in this vulnerable population.

**Supplementary Information:**

The online version contains supplementary material available at 10.1007/s11136-025-03920-3.

## Plain English Summary

This study examines how minimal hepatic encephalopathy (MHE), a common condition in patients with cirrhosis, affects thinking and coordination. High ammonia levels in the blood are linked to cognitive issues and coordination problems, which may impact patients' health-related quality of life (HRQoL). We studied 87 cirrhotic patients and 23 healthy volunteers, who completed tests to measure MHE, coordination, and HRQoL, along with blood tests for ammonia levels.

The results showed an association between higher ammonia levels and worse coordination in cirrhotic patients. However, these factors did not directly predict HRQoL. Interestingly, the study found that for women with MHE, high ammonia and poor coordination significantly affected their HRQoL, while this was not the case for men. These findings suggest that considering sex differences is crucial in understanding the way MHE impacts patients, which could improve diagnosis and treatment strategies.

## Introduction

Cirrhosis, a chronic liver disease that compromises liver function, poses a challenge to patient wellness and daily functioning [1], and could lead to hepatic encephalopathy (HE), a neuropsychiatric syndrome with symptoms ranging from cognitive and motor dysfunctions to severe coma [2, 3]. Around 30–50% of patients with liver cirrhosis show minimal hepatic encephalopathy (MHE), which represents the initial stage of HE [2, 3]. Although devoid of overt HE symptoms, these individuals exhibit subtle cognitive and motor deficits [4–10]. MHE generally does not interfere with basic daily activities such as dressing or eating [11], but is strongly associated with difficulties in executive function, eye-hand coordination (EHC), psychomotor speed, and sustained attention [7–10]. These deficits compromise the ability to perform more demanding tasks, such as managing finances, maintaining work performance, planning, and driving, which collectively have a detrimental impact on health-related quality of life (HRQoL) [12–16].

The adverse effects of MHE on quality of life are well-documented, particularly with respect to its association with reduced quality of life and increased work-related disability [11, 16, 17]. Previous studies have consistently reported lower scores across multiple HRQoL domains in patients with cirrhosis [18, 19]. Moreover, increasing severity of liver cirrhosis, or the presence of MHE, is closely linked to a decline in several components of HRQoL [15, 20]. Physical health is the most severely affected domain, underscoring the significant physical challenges faced by these patients [14, 21–23].

One key aspect of these physical limitations is the substantial impairment in eye-hand coordination (EHC) observed in patients with MHE [9, 10, 24]. EHC refers to the coordinated use of vision and hand movements to perform physical tasks, relying on the dynamic integration of multiple sensorimotor systems, including visual processing, attention, central neural processing, and motor function [25–27]. In MHE, these systems are significantly disrupted [10, 28–30], resulting in reduced functional independence. This impairment not only compromises the ability to perform daily tasks but also contributes to heightened frustration and diminished self-confidence, compounding the overall burden of MHE and further reducing HRQoL.

Beyond the physical impact, MHE also imposes a substantial mental and emotional toll. Psychiatric symptoms, including anxiety and depression, are well-documented comorbidities in patients with cirrhosis and contribute to a substantial decline in HRQoL [6, 11, 31]. These symptoms particularly impair emotional well-being and social functioning, and can intensify the physical limitations caused by cirrhosis complications, such as ascites or bleeding episodes, further compromising daily functioning [14, 32, 33].

Despite the established link between psychiatric symptoms and the broader context of cirrhosis, variability in study findings suggests underlying complexities that remain poorly understood. Emerging research indicates a potential correlation between HE and mood/anxiety disorders; however, current evidence is insufficient to draw definitive conclusions [34]. This indicates a critical knowledge gap and highlights the need for targeted interventions to address the unique challenges faced by this patient population.

Furthermore, hyperammonemia plays a crucial role in the pathogenesis of MHE, as ammonia is neurotoxic and readily crosses the blood–brain barrier [35–37]. Some studies show the synergistic effect of elevated serum ammonia levels and inflammation on neurological alterations in MHE patients [38, 39]. It is well-established that ammonia-mediated astrocyte dysfunction, neurotransmitter imbalances, and neuroinflammation collectively contribute to both motor and cognitive impairments associated with MHE [40, 41]. As mentioned, despite the link between MHE and a higher prevalence of neuropsychiatric symptoms such as depression, anxiety, and cognitive disorders [6, 11, 31], the mechanisms by which MHE contributes to subjective health outcomes of their emotional and physical well-being are not fully understood. While depression and anxiety are common in patients with cirrhosis, the specific ways in which MHE-related factors, such as hyperammonemia or motor coordination impairments, exacerbate these subjective experiences remain unclear. Addressing this gap is crucial for understanding the full impact of MHE on HRQoL.

Moreover, it is essential to assess sex disparities in this context. Specifically, chronic conditions like cirrhosis often exhibit differing epidemiology, natural progression, and treatment responses based on sex [42]. Examining potential sex-related distinctions is crucial for identifying individuals susceptible to psychological distress and cognitive decline. Prior studies have emphasized the adverse effects of chronic illnesses on HRQoL, with some findings suggesting that gender might influence perceptions of HRQoL in these conditions [43, 44]. Nevertheless, there is a paucity of literature on sex differences in cirrhosis, particularly regarding MHE. To redress this lack, therefore, this study aims to explore the potential associations between blood ammonia levels, EHC, and HRQoL as well as the moderating influence of sex on these associations in patients with and without MHE. The results of this study will enhance understanding of the negative effects of ammonia on quality of life and the influence of sex on these effects, enabling a more accurate evaluation of mental and physical health in cirrhotic patients.

## Methods

### Participants

This observational and cross-sectional study was reported following STROBE guidelines (Supplementary material). A total of 109 patients with liver cirrhosis were consecutively recruited from the outpatient clinics of Clínico and Arnau de Vilanova hospitals in Valencia, Spain. Diagnosis was based on clinical, biochemical, and ultrasonographic assessments. Inclusion criteria were chronic liver cirrhosis, ability to stand and walk unaided, and age over 18 years. Exclusion criteria were overt HE, recent alcohol intake (in the past six months), substance abuse history (excluding alcohol), antibiotic use, recent use of drugs affecting cognitive function (in the past 6 weeks), neurological or psychiatric disorders, and hepatocellular carcinoma.

A control group of 28 healthy volunteers was included in the study after liver disease was ruled out through clinical, analytical, and serological tests. The enrolled subjects exhibited no fever or any clinical or biological indications of recent infection.

Study participants were scheduled for two separate sessions: in the first session, blood was drawn, psychometric tests were performed to diagnose MHE, and subjects completed the SF-36 test. The coordination tests were administered during the second session held within the following three days.

Incomplete assessment in five controls and 22 patients left a final sample of 23 healthy volunteers and 87 patients (Supplementary Fig. 1).

Written informed consent was obtained from all participants before inclusion in the study. Study protocols were approved by the Research Ethics Committees of Hospital Clínico Universitario and Arnau de Vilanova Hospital of Valencia, Valencia, Spain (approval code: 2018/210; approval date: 2 March 2018) and were in accordance with the ethical guidelines of the Helsinki Declaration.

### MHE diagnosis

All participants underwent the Psychometric Hepatic Encephalopathy Score (PHES), a set of five psychometric tests utilized for MHE diagnosis [3, 6]. The PHES score, adjusted for age and educational level, was calculated using Spanish normality tables (http://www.redeh.org/TEST_phes.htm). Cirrhotic patients were categorized as having MHE if the score was ≤ − 4 points. According to the PHES score, patients were grouped into 24 individuals with MHE (18 men and 6 women), and 63 without MHE (NMHE) (49 men and 14 women). A group of 23 healthy volunteers (11 men and 12 women) were included as controls after performing the PHES battery to rule out any cognitive impairment (Table 1).

**Table 1:** Clinical and demographic variables of participants

	Controls (N = 23)	NMHE (N = 63)	MHE (N = 24)	*P*	*Effect size*
Sex (m/w)	11/12	49/14	18/6	0.025	0.265^b^
Age (years)^a^	63.7 ± 7.3	62.2 ± 8.6	62.6 ± 8.1	0.741	0.006^c^
Educational level^a^	14.1 ± 0.7	11.4 ± 0.4	11.8 ± 0.7	0.005	0.096^c^
Etiology of cirrhosis					
Alcohol	–	31	12		
HBV/HCV	–	2/11	1/2		
HCV + alcohol	–	0/2	1		
MASH/MASH + alcohol	–	9/4	7/1		
Other	–	4			
Child–Pugh class (A/B/C)		50/13/0	17/6/1		

NMHE and MHE, patients without and with minimal hepatic encephalopathy according to PHES score, *m* men, *w* women, *HBV* hepatitis B virus, *HCV* hepatitis C virus, *MASH* metabolic dysfunction-associated steatohepatitis, Child Pugh Scores measure the severity of chronic liver disease. Class C represents the highest classification of liver severity, while class A indicates the least severe. Reporting numbers represent the number of subjects in class A, B or C

^a^Values are expressed as mean ± standard deviation. Educational level is expressed as years of schooling

^b^Values are expressed as Cramér’s V coefficient

^c^Values are expressed as *n*_*p*_^*2*^

### Blood ammonia

Fasting venous blood ammonia levels were measured promptly after collection on the same day as psychometric testing using the PocketChemBA System Ammonia II Test Kit (Arkay, Inc., Kyoto, Japan) following the manufacturers’ instructions. This device employs microdiffusion technology to quantify ammonia concentrations in μM, using a 20 μL blood sample.

### Short form-36 health survey (SF-36)

HRQoL was measured using the Spanish version of the SF-36 [43]. It consists of 36 items related to different physical and mental health aspects, grouped into eight subscales: physical functioning, role-physical, bodily pain, general health, vitality, social functioning, role-emotional, and mental health. For each subscale, the items were transformed into a 0–100 scale, with higher scores indicating better HRQL. Following Hidalgo et al., 2021 [45], the eight subscales were stratified into two global measures: subjective physical health (SPH), which includes physical functioning, role-physical, bodily pain, general health, and vitality scales; and subjective mental health (SMH), which includes general health, vitality, social functioning, role-emotional, and mental health scales. In the present study, Cronbach's α was 0.89, indicating high internal scale reliability.

## Eye-hand coordination

### Vienna test system

The Motor Performance Series (MLS) from the Vienna Test System (version 8.0; Schuhfried, Austria) was used to assess four specific motor skills: aiming, linear tracking, tremor (steadiness), and tapping (see Supplementary methods). Following Zwierko et al. [46], indices were calculated as: Aiming Index (AI) = number of accurate hits per time taken for the test (nh/t); Linear Tracking Index (LTI) = number of errors per time taken for the test (ne/t); Steadiness Index (SI) = number of errors (ne); and Tapping Index (TI) = number of accurate hits (nh).

### Bimanual and visuomotor coordination tests

We conducted bimanual (BC) and visuomotor coordination (VC) tests as previously described [9]. Both tests were administered twice, without rest period, and the total time for completion was recorded in minutes [9].

Of the total participants, five (one MHE, two NMHE, and two controls) had missing data for all Vienna test outcomes (aiming, linear tracking, steadiness, and tapping), leaving 105 subjects included in these analyses, and a further 10 controls did not complete the SF-36, leaving 100 participants. For all other measures, the sample size remained at 110 participants.

### Statistical analysis

Group differences in all variables were analyzed using one-way ANOVA for continuous data and Chi-square test for categorical data.

To investigate whether blood ammonia levels were associated with EHC after adjusting for covariates (age, sex, educational level), we performed logistic regression analysis, including EHC as the dependent variable, covariates in block one, and ammonia levels in block two. To determine the relationship between ammonia levels and HRQoL, we next performed hierarchical regression analysis, including HRQoL variables (SPH or SMH) as the dependent variable, covariates in block one, and ammonia levels in block two. As the following step, the association between EHC and HRQoL was analyzed by again performing hierarchical regression analysis, including SPH or SMH as the dependent variable, the covariates in block one, and the EHC indices in block two.

We analyzed the influence of sex (men and women) and group (with or without MHE) on these relationships using moderated moderation analysis tested with the Hayes’ PROCESS macro (Model 3) for SPSS, which measures whether the moderating effect of one variable (W) on the relationship between the independent variable (X) and the dependent variable (Y) is further moderated by a second variable (Model 3; see Supplementary Fig. 2). A more detailed explanation is provided in Supplementary Methods. Age and educational level were added as covariates. All *p*-values reported are two-tailed, and the level of significance was set at *p* < 0.05. Statistical analyses were performed with SPSS 26.0 (IBM Corporation, Armonk, NY, USA).

A post hoc power analysis was conducted using G*Power software (version 3.1.9.7 for Windows) to calculate statistical power. Based on a moderate effect size (f^2^ = 0.15) and significance level (α = 0.05), with a total sample size of n = 87, the power analysis yielded 1 − *β* = 0.811. See Supplementary Methods for a more detailed explanation.

To assess the potential bias introduced by missing data, we compared results with and without the excluded participants. These analyses confirmed that excluding subjects with missing data did not alter the main results of the study (Supplementary Table 4).

## Results

### Preliminary analyses

Descriptive statistics of each parameter and differences between MHE patients, NMHE patients and the control group are presented in Table 1.

MHE patients showed significantly higher blood ammonia levels than controls (*p* = 0.003) but no significant differences were found between MHE and NMHE patients (Table 2). When separating men and women within each group, overall ANOVA revealed no statistically significant sex-dependent differences in ammonia levels (*p* = 0.064 for men, *p* = 0.075 for women) (Supplementary Table 1). However, pairwise comparisons showed significantly higher blood ammonia levels in both men and women with MHE compared to their respective controls (*p* = 0.002 and *p* = 0.01, respectively).

**Table 2:** Blood ammonia levels, performance in eye-hand coordination tests, and SF-36 scores in the three study groups

	Controls (N = 23)	NMHE (N = 63)	MHE (N = 24)	*F (df, RSE)*	*G*lobal anova *p*	*Effect size (N*_*P*_^*2*^)
Blood ammonia (µM)	11.86 ± 5.8	27.85 ± 3.5	39.22 ± 5.7^**^	**5.81 (2, 107)**	**0.004**	0.09
Vienna Test System						
Aiming index (nh/t)	1.98 ± 0.1	1.66 ± 0.1^**^	1.18 ± 0.1^*** ααα^	20.61 (2, 102)	**0.001**	0.288
Linear tracking index (ne/t)	0.61 ± 0.2	0.31 ± 0.1	0.55 ± 0.2	0.79 (2, 104)	0.456	0.015
Steadiness index (ne)	9.85 ± 3.4	18.89 ± 2.0	28.52 ± 3.2^*** α^	8.08 (2, 102)	**0.001**	0.137
Tapping index (nh)	187.9 ± 5.3	172.9 ± 3.1	148.1 ± 5.1^*** ααα^	15.20 (2, 102)	**0.001**	0.230
Coordination Tests						
Visuomotor coordination (min)	2.44 ± 0.14	2.80 ± 0.08	4.04 ± 0.13^*** ααα^	41.57 (2, 107)	**0.001**	0.437
Bimanual coordination (min)	2.09 ± 0.14	2.30 ± 0.9	3.36 ± 0.14^*** ααα^	25.65 (2, 107)	**0.001**	0.324
SF-36 Test						
Subjective physical health	254.1 ± 39.0	315.4 ± 17.8	252.2 ± 28.9	2.27 (2, 97)	0.108	0.045
Subjective mental health	226.8 ± 38.1	335.6 ± 17.3^*^	282.5 ± 28.1	**3.945 (2, 97)**	**0.023**	0.075

Values are expressed as mean ± SEM. NMHE and MHE, patients without and with minimal hepatic encephalopathy according to PHES score, *df* degrees of freedom, *RSE* residual standard error, *n*_*p*_^*2*^ partial eta squared, *t* time taken for the test, *ne* number of errors, *nh* number of hits, *min* total minutes employed in the tests. Significant differences compared to controls are indicated by asterisks: *p < 0.05; **p < 0.01; ***p < 0.001; and from NMHE patients by α. (α: *p* < 0.05; ***/ααα: *p* < 0.001). Significant *P* values (p < 0.05) are in bold

Regarding the aiming index (AI) from the Vienna test, MHE patients performed poorly compared with either NMHE patients (*p* < 0.001) or controls (*p* < 0.001), and NMHE patients showed significantly worse performance than controls (*p* = 0.009). Similar differences were observed in SI and TI, in which MHE patients showed higher number of errors and lower hits, respectively, than either NMHE patients (*p* = 0.036 and *p* < 0.001, respectively) or controls (*p* < 0.001), without significant differences between NMHE and controls. Additionally, no significant between-group differences were found in LTI (Table 2).

In visuomotor and bimanual coordination tests, MHE patients also showed poor performance compared with NMHE patients (*p* < 0.001) and controls (*p* < 0.001), without significant differences between NMHE and controls (Table 2).

Regarding HRQoL, no significant between-group differences were found in SPH. In SMH, there were no significant differences between MHE patients and NMHE patients or controls, but NMHE patients reported significantly better SMH than controls (*p* = 0.033) (Table 2).

### Relationship between ammonia levels and EHC indices

Results showed that higher ammonia levels were associated with worse AI (*p* = 0.003) TI (*p* = 0.040), and SI (*p* = 0.006) and longer execution time in BC (*p* = 0.025). However, LTI and VC were not linked with blood ammonia (Table 3).

**Table 3:** Relationship between blood ammonia levels and eye-hand coordination indices

	Δ*R*^2^	*B*	*p*
Aiming index	0.165		
Ammonia		− 0.296	**0.003**
Sex		− 0175	0.063
Educational level		0.071	0.447
Age		− 0.273	**0.004**
Linear tracking index	0.007		
Ammonia		− 0.130	0.188
Sex		− 0.023	0.814
Educational level		0.102	0.301
Age		0.044	0.657
Steadiness index	0.052		
Ammonia		0.273	**0.006**
Sex		− 0.109	0.275
Educational level		− 0.096	0.968
Age		− 0.016	0.870
Tapping index	0.157		
Ammonia		− 0.190	**0.040**
Sex		0.034	0.717
Educational level		0.157	0.091
Age		− 0.343	**0.001**
Visuomotor coordination	0.122		
Ammonia		0.114	0.217
Sex		0.162	0.082
Educational level		− 0.137	0.141
Age		0.290	**0.002**
Bimanual coordination	0.145		
Ammonia		0.206	**0.025**
Sex		0.100	0.277
Educational level		− 0.161	0.081
Age		0.284	**0.002**

*∆R*^2^ R square change. Significant *P* values (p < 0.05) are in bold

### Relationship between ammonia and HRQoL: the role of sex

To test associations between ammonia and both SPH and SMH, linear regression analyses were performed. In Model 1, sex was a significant predictor for both SPH (*p* = 0.008) and SMH (*p* < 0.001) (Table 4). Age and educational level showed no significant relationships with SPH or with SMH (Table 4). Model 2 incorporated blood ammonia levels, but no significant changes were observed in the explained variance for either SPH (∆R^2^ = 0.000) or SMH (∆R^2^ = 0.001). The regression coefficients for ammonia were not significant for either dimension of HRQoL (Table 4).

**Table 4:** Relationship between blood ammonia and subjective physical and mental health across all groups

	Subjective Physical Health				Subjective Mental Health			
	R^2^	∆ R^2^	B	*p*	R^2^	∆ R^2^	B	*p*
Model 1	0.269	0.072**			0.33	0.11***		
Age			0.055	0.589			0.132	0.183
Sex			0.271	**0.008**			0.337	** < 0.001**
Educational level			− 0.003	0.977			− 0.088	0.366
Model 2	0.270	0.000			0.338	0.001		
Ammonia			− 0.017	0.866			0.025	0.793

*R*^2^ R square, *∆R*^2^ R square change. **p < 0.01; *** p < 0.001 Significant *P* values (p < 0.05) are in bold

**Fig. 1:**
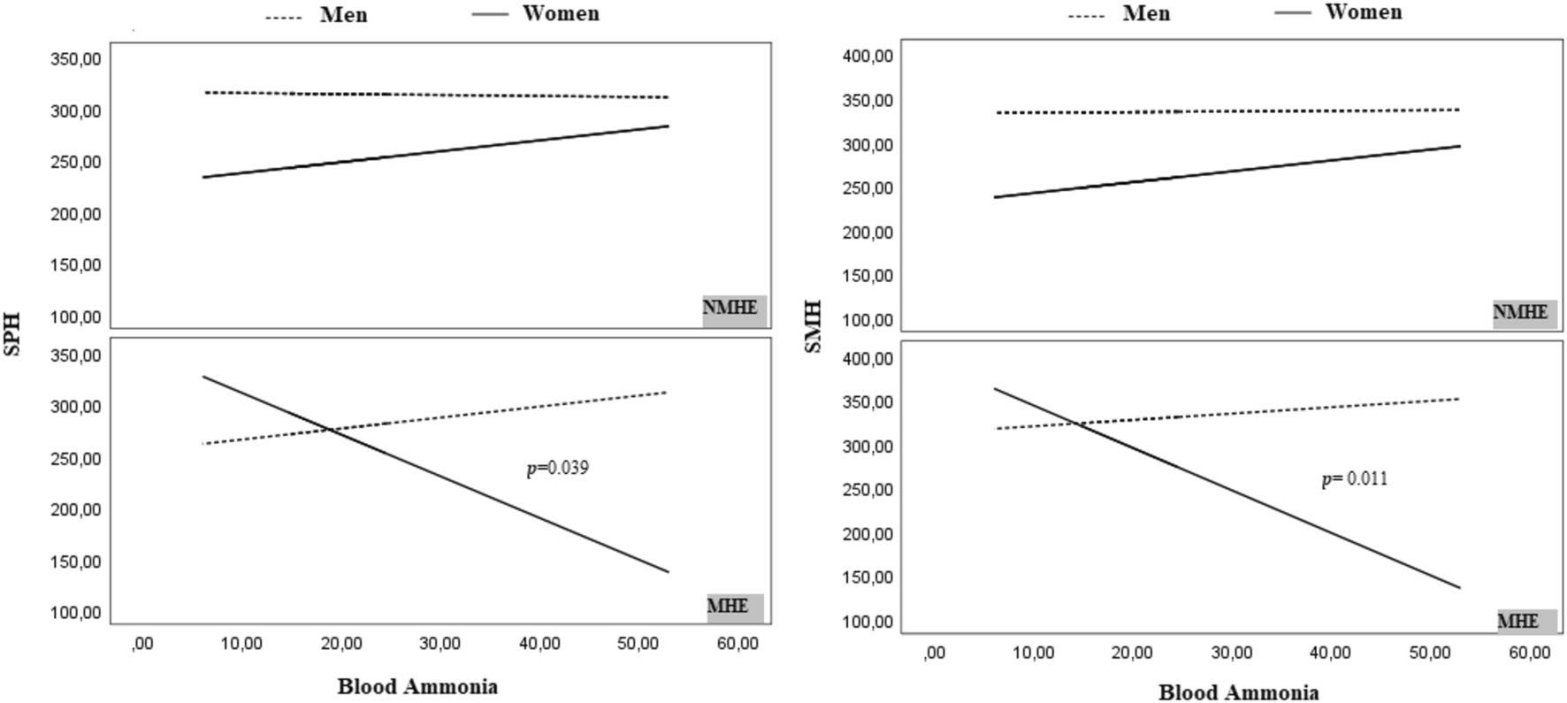
Relationship between health-related quality of life (HRQoL) and blood ammonia in cirrhotic patients classified by sex. The Y axis represents the total SPH or SMH values and the X axis the blood ammonia levels in both groups. While significant negative associations were observed only in women with MHE, no significant relationships were found in the NMHE group for SMH. NMHE, MHE, patients without and with minimal hepatic encephalopathy, respectively; SPH, subjective physical health; SMH, subjective mental health

However, the moderated moderation analysis revealed significant X × W × Z interactions between ammonia levels and SPH (*p* = 0.016) (Table 5; Fig. 1). Specifically, higher ammonia levels were associated with lower SPH, but this effect was only significant in women with MHE (*p* = 0.039). No significant association between blood ammonia levels and SPH was observed in the NMHE, regardless of sex. As regards SMH, similar X × W × Z interactions were found (*p* = 0.005), significant only in women with MHE (*p* = 0.011). No significant association was observed for SMH in the NMHE group, independently of sex (Table 5, Fig. 1 and Supplementary Table 2).

**Table 5:** Moderated moderation analysis between blood ammonia levels and subjective physical and mental health in MHE and NMHE groups

X variable: Ammonia levels							
Y variable	effect		statistics				
SPH	Δ*R*^2^ interaction = 0.058 *F* = 6.031, df (1,88) = 179.21 *p* = 0.016 LLCI = 34.19 ULCI = 324.224						
			*B*	SE	t	*p*	*CI 95%*
	Interactions						
	Test of conditional ammonia X group by sex						
		Men	30.613			0.381	
		Women	− 148.596			**0.023**	
	Conditional effect of SPH depending on sex and group						
	Without MHE	Men	− 1.942	17.095	− 0.114	0.910	− 35.91, 32.03
	Without MHE	Women	29.015	39.242	0.739	0.462	− 48.97, 107.00
	MHE	Men	28.671	33.077	0.867	0.388	− 37.06, 94.40
	MHE	Women	− **119.581**	**56.903**	− **2.102**	**0.039**	− **232.66, **− **6.50**

**SMH**	Δ*R*^2^ interaction = 0.071 *F* = 8.026, df (1,88) = 6.709 *p* = 0.005, LLCI = 2.003 ULCI = 11.415						
			*B*	SE	**t**	*p*	*CI 95%*
	Interactions						
	Test of conditional ammonia X group by sex						
		Men	0.457			0.686	
		Women	− 6.252			**0.003**	
	Conditional effect of SMH depending on group and sex						
	Without MHE	Men	0.076	0.555	0.137	0.891	− 1.03, 1.18
	Without MHE	Women	1.433	1.274	1.126	0.263	− 1.10, 3.96
	MHE	Men	0.533	1.073	0.496	0.621	− 1.60, 2.67
	**MHE**	**Women**	− **4.819**	**1.847**	− **2.610**	**0.011**	− **8.49, **− **1.15**

*∆R*^2^ R square change, *HRQoL* health-related quality of life, *CI* confidence interval, *MHE* minimal hepatic encephalopathy, *LLCI* lower limit confidence interval, *ULCI* upper limit confidence interval, *SPH* subjective physical health, *SMH* subjective mental health, *SE* standard error. Significant *P* values (p < 0.05) are in bold

### Relationship between EHC and HRQoL in cirrhotic patients: the role of sex

Linear regression analyses showed no associations between EHC outcomes with SPH or SMH (Table 6). However, results of moderated moderation analysis revealed significant X × W × Z interactions across several motor EHC outcomes with SPH and SMH (Table 7 and Supplementary Table 3).

**Table 6:** Relationship between hand–eye coordination and subjective physical and mental health

	Subjective Physical Health				Subjective Mental Health			
	R^2^	∆ R^2^	B	*p*	R^2^	∆ R^2^	B	*p*
Model 1	0.271	0.073			0.342	0.117		
Age			− 0.039	0.713			0.042	0.680
Sex			0.271	**0.009**			0.342	**0.001**
Educational level			− 0.014	0.893			− 0.107	0.283
Model 2	0.305	0.093			0.359	0.129		
Aiming Index			− 0.059	0.610			0.032	0.748
Linear Tracking Index			− 0.040	0.716			− 0.026	0.792
Steadiness Index			− 0.067	0.546			− 0.038	0.707
Tapping Index			0.004	0.971			− 0.018	0.858
Visuomotor coordination			− 0.106	0.482			0.012	0.904
Bimanual coordination			0.123	0.378			0.077	0.441

*R*^2^ R square, *∆R*^2^ R square change

**Table 7:** Moderated moderation analysis between hand–eye coordination outcomes and subjective physical and mental health in MHE and NMHE groups

X variable: Aiming index								
				***B***	**SE**	**t**	***p***	***CI 95%***
**SPH**	Δ*R*^2^ interaction = 0.0333 *F* = 3.368, df (1,86) = − 10.611 *p* = 0.070 LLCI = − 22.103 ULCI = 0.882							
	Without MHE	Men		0.413	3.409	0.121	0.904	− 6.363, 7.189
	Without MHE	Women		− 1.082	2.083	− 0.520	0.605	− 5.22, 3.059
	MHE	Men		6.391	5.464	1.170	0.245	− 4.471, 17.252
	MHE	Women		− 5.716	3.496	− 1.635	0.106	− 12.665, 1.234
**SMH**	Δ*R*^2^ interaction = 0.0392 *F* = 4,189, df (1,86) = − 11.210 *p* = 0.044 LLCI = − 22.096 ULCI = − 0.322							
	Without MHE	Men		− 0.048	1.9737	− 0.024	0.981	− 3.971, 3.876
	**Without MHE**	**Women**		2.0504	3.2292	-0.635	0.527	− 4.369, 8.470
	MHE	Men		− 4.3137	3.3117	− 1.302	0.196	− 10.987, 2.269
	MHE	Women		8.9936	5.1760	1.737	0.086	− 1.296, 19.283

X variable: Linear tracking index								
				***B***	**SE**	**t**	***p***	***CI 95%***
**SPH**	Δ*R*^2^ interaction = 0.051 *F* = 5.501, df (1,89) = − 337.184 *p* = 0.021 LLCI = − 622.617 ULCI = − 51.750							
	Without MHE	Men		4.886	19.053	0.256	0.798	− 32.97, 42.74
	**Without MHE**	**Women**		− **361.314**	**137.441**	− **2.629**	**0.010**	− **634.41, **− **88.22**
	MHE	Men		13.659	47.606	0.287	0.775	− 80.93, 108.25
	MHE	Women		− 15.357	17.354	− 0.885	0.379	− 49.84, 19.13
**SMH**	Δ*R*^2^ interaction = 0.050 *F* = 5.623, df (1,89) = − 326.73 *p* = 0.020 LLCI = − 600.764 ULCI = − 50.699							
	Without MHE	Men		4.528	18.292	0.248	0.805	-31.82, 40.87
	**Without MHE**	**Women**		− **357.076**	**131.951**	− **2.706**	**0.008**	− **619.26,**− **94.89**
	MHE	Men		24.222	45.704	0.530	0.598	− 66.59, 115.03
	MHE	Women		− 10.651	16.661	− 0.639	0.524	− 43.76, 22.45

X variable: Steadiness index								
**SPH**	Δ*R*^2^ interaction = 0.045 *F* = 4.800, df (1,84) = 8.543, *p* = 0.031, LLCI = 0.792 ULCI = 16.293							
	Without MHE	Men		0.098	1.048	0.093	0.926	-1.99, 2.18
	Without MHE	Women		2.002	2.262	0.885	0.379	-2.49, 6.50
	MHE	Men		− 2.050	1.743	− 1.177	0.243	− 5.52, 1.42
	**MHE**	**Women**		− **8.689**	**3.265**	− **2.661**	**0.009**	− **15.18, **− **2.20**
**SMH**	Δ*R*^2^ interaction = 0.063 *F* = 7.294, df (1,84) = 9.833, *p* = 0.008, LLCI = 2.593 ULCI = 17.074							
	Without MHE	Men		0.0057	0.979	0.006	0.996	− 1.94, 1.95
	Without MHE	Women		1.623	2.114	0.768	0.445	− 2.58, 5.83
	MHE	Men		− 1.629	1.628	− 1.001	0.320	− 4.87, 1.61
	**MHE**	**Women**		− **9.856**	**3.051**	− **3.228**	**0.002**	− **15.91, **− **3.78**

**X variable: Tapping index**								
**SPH**	Δ*R*^2^ interaction = 0.0566 *F* = 6.211, df (1,84) = − 7.698, *p* = 0.015, LLCI = − 13.841 ULCI = − 1.556							
	Without MHE	Men		− 0.320	0.617	− 0.519	0.605	− 1.55, 0.91
	Without MHE	Women		− 2.593	1.655	− 1.567	0.121	− 5.88, 0.70
	MHE	Men		1.547	0.903	1.714	0.090	− 0.25, 3.34
	**MHE**	**Women**		**6.973**	**3.264**	**2.136**	**0.036**	**0.48, 13.46**
**SMH**	Δ*R*^2^ interaction = 0.063 *F* = 7.869, df (1,87) = − 7.856, *p* = 0.006, LLCI = − 13.423 ULCI = − 2.290							
	Without MHE	Men		− 0.325	0.561	− 0.579	0.564	− 1.44, 0.79
	Without MHE	Women		− 2.589	1.532	− 1.691	0.095	− 5.63, 0.46
	MHE	Men		1.434	0.828	1.732	0.086	− 0.21, 3.08
	**MHE**	**Women**		**7.027**	**2.965**	**2.369**	**0.020**	**1.13, 12.92**

X variable: Visuomotor coordination								
**SPH**	Δ*R*^2^ interaction = 0.0312 *F* = 3.369, df (1,88) = -8.975 *p* = 0.069, LLCI = -19.814 ULCI = 1.865							
	Without MHE	Men		25.660	31.915	0.8040	0.423	− 37.764, 89.086
	Without MHE	Women		108.405	58.607	18.497	0.068	− 8.065, 224.874
	MHE	Men		− 22.821	28.416	− 0.8031	0.424	− 79.293, 33.650
	MHE	Women		− 104.091	73.971	− 14.072	0.163	− 251.093, 42.911
**SMH**	Δ*R*^2^ interaction = 0.0469 *F* = 5.2436, df (1,88) = 195.936, *p* = 0.024 LLCI = 25.892 ULCI = 365.979							
	Without MHE	Men		11.576	30.560	0.379	0.706	− 49.16, 72.31
	Without MHE	Women		91.905	56.120	1.638	0.105	− 19.62, 203.43
	MHE	Men		− 22.437	27.211	− 0.825	0.412	− 76.51, 31.64
	MHE	Women		− 138.044	70.832	− 1.949	0.055	− 278.81, 2.72

X variable: Bimanual coordination								
			***B***		**SE**	**t**	***p***	***CI 95%***
**SPH**	Δ*R*^2^ interaction = 0.077 *F* = 8.997, df (1,88) = 431.142, *p* = 0.004 LLCI = − 145.490 ULCI = 716.784							
	**Without MHE**	**Men**		**90.226**	**38.853**	**2.322**	**0.023**	**13.01, 167.44**
	**Without MHE**	**Women**		**177.155**	**85.918**	**2.062**	**0.042**	**6.41, 347.90**
	MHE	Men		15.475	21.263	0.728	0.469	− 26.78, 57.73
	**MHE**	**Women**		− **328.737**	**128.481**	− **2.559**	**0.012**	− **584.07, **− **73.41**
**SMH**	Δ*R*^2^ interaction = 0.059 *F* = 7.035, df (1,88) = − 371.922 *p* = 0.010 LLCI = 93.212 ULCI = 650.573							
			***B***		**SE**	**t**	***p***	***CI 95%***
	**Without MHE**	**Men**		**81.401**	**37.903**	**2.148**	**0.035**	**6.07, 156.73**
	Without MHE	Women		146.223	83.815	1.745	0.085	− 20.34, 312.79
	MHE	Men		5.179	20.336	0.249	0.803	− 36.04, 46.40
	**MHE**	**Women**		− **301.921**	**125.336**	− **2.409**	**0.018**	− **551.00, **− **52.84**

*R*^2^ R square, *∆R*^2^ R square change, *SPH* subjective physical health, *SMH* subjective mental health, *AI* aiming index, *LTI* linear tracking index, *SI* steadiness index, *TI* tapping index, *VC* visuomotor coordination, *BC* bimanual coordination, *LLCI* lower limit confidence interval, *ULCI* upper limit confidence interval. **p < 0.01; *** p < 0.001

Regarding the LTI, women without MHE exhibited pronounced negative effects (*p* = 0.010 for SPH; *p* = 0.008 for SMH), whereas there was no significant association between LTI and SPH or SMH in the MHE group, independently of sex.

For the SI**,** associations were significant only in women with MHE (*p* = 0.009 for SPH; *p* = 0.002 for SMH). In contrast, no significant associations were observed in the NMHE group, regardless of sex.

As regards the TI, women with MHE exhibited positive interactions (*p* = 0.036 for SPH; *p* = 0.020 for SMH). No significant associations were found in the NMHE group.

In terms of BC, the direction of the interaction changed depending on the group and sex. For SPH, the relationship was positive in men (*p* = 0.023) and women (*p* = 0.042) without MHE, but negative in women with MHE (*p* = 0.012). For SMH, the relationship was positive in men without MHE (*p* = 0.035), but negative in women with MHE (*p* = 0.018).

No significant associations were found for the AI or VC with SPH (Table 7). For SMH, a significant X × W × Z interaction was found (AI: *p* = 0.044; VC: *p* = 0.024). However, post hoc analyses revealed that the conditional effects of VC or AI within specific groups (MHE and without MHE) and by sex did not reach statistical significance.

## Discussion

The present study was aimed at exploring the relationship between blood ammonia levels, eye-hand coordination, minimal hepatic encephalopathy, and health-related quality of life, while also investigating the moderating influence of sex on these associations.

Our study revealed a significant association between elevated blood ammonia levels and impaired EHC performance among cirrhotic patients. Specifically, higher blood ammonia concentrations correlated with worse outcomes across multiple EHC tasks, including aiming, tapping, steadiness, and bimanual coordination. Furthermore, patients with MHE exhibited notably lower performance across most EHC tasks compared to patients without MHE and the control group.

Previous studies have highlighted slowness and inaccuracy in similar tasks, underscoring the disruption of motor control preceding physical frailty and reduced coordination among MHE patients [24, 47]. Other previous research, while not focusing specifically on EHC, has demonstrated increased variability in gripping force with the right hand and lateral pinch with both hands in patients with MHE [10, 48]. Further variations in motor performance also observed include increased variability in motor reaction times among MHE patients.

Blood ammonia levels play a crucial role in liver cirrhosis, as raised levels can lead to substantial neurological difficulties including motor impairment. Prior research in animal models of HE has underscored the impact of hyperammonemia in various neuronal areas, suggesting its contribution to the decline in motor function observed in cirrhotic patients with MHE [49–51]. To our best knowledge, however, this study is among the first to explore this relationship in the specific context of fine motor skills and their implications in patient-perceived quality of life.

Interestingly, no significant associations of blood ammonia levels or EHC were found with either SPH or SMH. However, a significant interaction emerged when including sex as a moderator in the analysis. These findings suggest that while blood ammonia levels and EHC may not directly influence SPH or SMH in MHE patients, underlying factors such as sex may moderate the relationship between these variables and HRQoL, indicating that this relationship may be sex-specific.

The moderated moderation analysis revealed that this relationship was significant in women with MHE. Specifically, elevated ammonia levels were associated with lower scores in the SPH and SMH dimensions. From a biological perspective, women have been documented to exhibit greater susceptibility to neurocognitive alterations and higher comorbidity rates, likely due to differences in hepatic and cerebral metabolism [52]. Estrogen and other sex hormones have been shown to influence the neuroinflammatory response and astrocyte function, exacerbating the effects of ammonia accumulation in the brain [53, 54].

In our postmenopausal sample, however, the role of these hormones likely reflects the downstream effects of reduced estrogenic signaling rather than direct hormonal activity. This impaired estrogenic signaling, compounded by the absence of other factors critical for neuronal health, may amplify vulnerability to ammonia-induced astrocyte dysfunction [55]. Such dysfunction disrupts cerebral homeostasis and appears to result in greater neuropsychiatric impairments in women than in men. These findings highlight the importance of considering estrogen not as a primary contributor but as part of a complex cascade of interrelated factors that collectively lead to the neuropsychiatric impairments observed in this demographic. Additionally, hormonal fluctuations may further exacerbate the perception of fatigue and weakness, significantly impacting subjective quality of life in women.

Women with MHE also demonstrated stronger associations between some EHC deficits and poorer HRQoL outcomes compared to men, suggesting that women may be more sensitive to motor impairments. This finding is consistent with prior research suggesting that chronic conditions have a greater impact on HRQoL in women, possibly due to hormonal, inflammatory, or psychosocial factors [56–59]. However, the associations between EHC and HRQoL were not consistent across all indices. Significant relationships between certain indices (e.g., tapping, steadiness and bimanual coordination) and both dimensions of HRQoL (SPH and SMH) were observed in women with MHE but not in men or non-MHE patients, whereas other indices, such as aiming and visuomotor coordination, showed no significant associations with HRQoL in women with MHE. These inconsistencies may reflect a varying sensitivity of EHC to the motor and cognitive deficits that impact HRQoL. Steadiness**,** tapping**,** and bimanual coordination assess fine motor skills and coordination, essential for performing daily activities that require successful spatial–temporal interactions [60, 61]. In contrast, visuomotor tracking tasks, (aiming and linear tracking) involve combining visual perception with motor execution such as following a moving target. They require continuous perceptual-motor regulation, and this regulation involves prospective control of action, based on exploiting control laws that minimize the gap between the actual behavior and the desired one [62–64]. For instance, tapping, steadiness and bimanual coordination may capture fine motor control and stability under high cognitive demand, which may be more directly relevant to daily functioning and perceived quality of life in women.

A number of factors may help explain our observations. Women may rely more on this kind of fine motor skills in their daily activities if performing societally assigned roles such as caregiving or household tasks. As a result, deficits in these areas might appear more pronounced or distressing in women than men, affecting both their performance and perceived quality of life. Fine motor tasks like steadiness, tapping, and bimanual coordination demand precise control, stability, and coordination, which are sensitive to the subtle motor and cognitive disruptions caused by MHE. Women with MHE may exhibit greater impairments in these tasks due to sex-specific vulnerabilities in neural processing, heightened sensitivity to inflammation, and the interaction between motor and cognitive deficits. This heightened vulnerability may stem from biological factors that also make women more responsive to the detrimental cognitive and behavioral effects of chronic inflammation [65], underscoring the importance of considering sex differences when evaluating the broader impacts of MHE.

We also found significant interaction effects for linear tracking in women without MHE, who showed pronounced negative associations between this outcome and both SPH and SMH. These findings indicate that even in the absence of MHE, motor coordination deficits such as those measured by linear tracking may affect women’s perception of their overall quality of life. It suggests that difficulties in tasks requiring precise visuomotor tracking may interfere with daily activities and psychosocial functioning.

Our results agree with previous studies showing gender disparities in the impact of chronic conditions on quality of life [56–59], but the factors which may explain this relationship are yet to be pinpointed. Specifically, women with chronic health conditions seem to report and experience poorer quality of life in the physical and psychological domains compared to men with chronic health conditions. Societal and cultural factors may contribute to this gender-specific association. Women may encounter distinct psychosocial stressors or utilize coping mechanisms differently from men, potentially impacting their perception and reporting of HRQoL in the context of cirrhosis. Particularly, societal expectations concerning gender roles may heighten this difference, as women often assume caregiving and household management responsibilities, which heavily rely on fine motor skills. Lastly, differences in coping mechanisms and responses to illness between genders may further elucidate the varying impact of EHC on quality of life perception.

Finally, an unexpected finding that poorer bimanual coordination performance (indicated by longer execution times) is associated with better SPH and SMH health in men without MHE warrants careful interpretation. This counterintuitive result suggests that factors beyond motor execution directly influence men’s perceptions of their HRQoL. Men may perceive slower task completion as caused by task complexity rather than personal limitations, framing it positively as evidence of persistence and effort. This mindset, emphasizing quality over speed, may mitigate negative emotional impacts and reinforce a sense of control. Additionally, slower coordination may not meaningfully affect functional independence in daily life, allowing men to maintain a positive view of their health despite worse performance. Future studies should investigate how men interpret motor performance and its relevance to their daily lives. Understanding these interpretations can help clarify the mechanisms driving this paradoxical relationship.

Overall, our study is not without limitations. Firstly, the sample size of cirrhotic patients, particularly women, was somewhat reduced compared to men and healthy volunteers. This could limit the generalizability of the findings; larger and more balanced samples, particularly in terms of sex distribution among cirrhotic patients, would provide a more robust basis for drawing conclusions. Nonetheless, it is important to note that this unbalanced number of women and men reflects the different prevalence of this illness between the two sexes. Another potential drawback is the lower smaller sample size of the control group than the patient group, although control subjects were age-matched with patients. Secondly, HRQoL assessment was based solely on self-report measures using the SF-36 questionnaire. While widely used and validated, self-reported measures may be influenced by various factors such as mood, perception, and cognitive biases. Incorporating objective measures like waist circumference, HRV, blood pressure, triglyceride levels, etc., or clinician-rated assessments of HRQoL, could offer more comprehensive insight. Thirdly, the study focused on hyperammonemia and EHC as predictors of HRQoL, but other potential confounding variables, such as comorbidities, medication use, socioeconomic status, and psychological factors, were not comprehensively assessed or controlled for in the analyses. Considering these factors in future studies could provide a more nuanced understanding of the determinants of HRQoL. Finally, longitudinal measures were not included, so causality between ammonia levels, motor dysfunction, and HRQoL cannot be established. Future research should include long-term follow-up to evaluate how fluctuations in ammonia levels affect the progression of motor symptoms and quality of life. Additionally, the inclusion of additional biomarkers such as systemic inflammation could shed more light on underlying pathophysiological mechanisms.

## Conclusions

This study contributes significantly to the understanding of MHE and its impact on HRQoL in cirrhotic patients, by exploring the associations between hyperammonemia, EHC, and HRQoL while considering sex differences and MHE status. The findings of this study suggest that sex moderates the relationship between ammonia levels, EHC deficits, and HRQoL. Women with MHE are disproportionately affected, showing greater sensitivity to motor impairments and their impact on HRQoL. These impairments are evident in tasks requiring fine motor skills, such as precision, steadiness, and bimanual coordination, which are essential for daily functioning. The study underscores the importance of considering sex as a critical factor when evaluating neurological and quality-of-life outcomes in patients with MHE, highlighting the importance of considering biological, psychological, and social factors in the assessment and management of this population. Future research should address these disparities by incorporating larger, more diverse samples and exploring additional biomarkers to better understand the underlying mechanisms and inform targeted interventions. Future longitudinal studies with larger, more balanced samples are needed to confirm these findings and explore their clinical implications.

## Supplementary Information

Below is the link to the electronic supplementary material.

Supplementary file1 (DOCX 123 KB)

## Data Availability

The authors declare that all the data supporting the findings of this study are available within this article and Supporting Material.
