# Supplementary material for: Blood ammonia and eye-hand coordination negatively affect health-related quality of life in women with minimal hepatic encephalopathy
Source: Qual Life Res. 2025 Apr 1;34(6):1669–82. doi: 10.1007/s11136-025-03920-3 (PMC12119665; doi:10.1007/s11136-025-03920-3)
Supplement: Supplementary file 1 — Supplementary file1 (DOCX 123 KB) [file 11136_2025_3920_MOESM1_ESM.docx]

***Quality of Life Research***

**SUPPLEMENTARY MATERIALS**

**Blood ammonia and eye-hand coordination negatively affect health-related quality of life in women with minimal hepatic encephalopathy**

Daniela Batallas, Juan José Gallego, Franc Casanova-Ferrer, Alessandra Fiorillo, Pablo Rivas-Diaz, Adrià López, Yaiza-María Arenas, Luis Aparicio, Desamparados Escudero-García, Lucía Durbán, María-Pilar Rios, Salvador Benlloch, Amparo Urios, Vanesa Hidalgo, Carmina Montoliu^*^, Alicia Salvador

*Corresponding author: Carmina Montoliu. Department of Pathology, Faculty of Medicine, University of Valencia, 46010 Valencia, Spain. E-mail: carmina.montoliu@uv.es

**Contents**

- **Supplementary Methods**:
  - Bimanual and visuomotor coordination tests
  - Vienna Test System
  - Statistical power calculation
  - Moderated moderation model
- **Supplementary Figure 1.** Flow chart of the steps followed in the selection of the study sample
- **Supplementary Figure 2.** Moderated moderation model
- **Supplementary Table 1.** Blood ammonia levels according to sex in the three study groups
- **Supplementary Table 2.** Statistics of the interaction of ammonia levels in moderated moderation analysis
- **Supplementary Table 3.** Statistics of the interaction of EHC indices in moderated moderation analysis
- **Supplementary Table 4.** Complete sample blood ammonia levels, performance in eye-hand coordination tests, and SF-36 scores in the three study groups
- **STROBE Statement**—Checklist of items that should be included in reports of cross-sectional studies

**SUPPLEMENTARY METHODS**

**Visuomotor and bimanual coordination tests**

The visuomotor coordination test was conducted using a board featuring a grid of uniform perforations arranged in six rows and columns. The perforations are aligned yet oriented differently, with a corresponding set of metal pieces that fit precisely into the holes. Participants are required to place these pieces in rows with their dominant hand until the board is entirely filled. The test is administered twice, without a rest period, and the total time for completion is recorded in minutes, which serves as a key measure in this study [9].

The other test employed was bimanual coordination, which entails relocating a series of metallic pegs from one half of a pegboard to the other. This task requires symmetrical and simultaneous movements with both hands. The procedure is repeated twice in each direction, without rest period, and the time taken for completion was recorded in minutes for this study [9].

Participants performed each trial consecutively without breaks.

**Vienna Test System**

The Motor Performance Series (MLS) from the Vienna Test System (version 8.0; Schuhfried, Austria) was used to assess four specific motor skills: aiming, linear tracking, tremor (steadiness), and tapping. The MLS consists of a standardized panel (300×300×15 mm) with holes and contact fields for various subtests. One of two pens (according to the subject’s dominant hand) was used to evaluate motor skills. The system recorded the number and duration of contacts between the pen and board as electrical closures (5 V, 20 mA). Tasks included touching 20 sensors in a row for aiming; following a groove with bends, angles and curves for linear tracking; keeping the pen inside a 5 mm hole for 32 seconds for tremor steadiness; and tapping a 40 × 40 mm square as many times as possible within 32 seconds for hand tapping.

Once these visuomotor and bimanual coordination tests were completed, participants proceeded directly to the tasks of the Vienna Test System, which were administered in the following prescribed order: Tremor, Linear Tracking, Aiming, and Tapping. This sequence was consistent across all participants to ensure standardization.

**Statistical power calculation**

A post hoc power analysis was conducted using the G*Power software (version 3.1.9.7 for Windows). The analysis was designed to evaluate the fixed-effects multiple linear regression model, specifically to detect a significant increase in R^2^ associated with the inclusion of four main predictors (X, X×W, X×Z and X×W×Z) in a model that controlled for two additional covariates (age and educational level). Based on a moderate effect size (f^2^=0.15) and significance level (α=0.05), with a total sample size of n=87, the power analysis yielded 1−*β*=0.811.

**Moderated moderation model**

Hayes’ PROCESS Macro (Model 3) was used to test for moderated moderation, to determine whether the moderating effect of one variable (W) on the relationship between the independent variable (X) and the dependent variable (Y) would be further moderated by a second variable (Model 3; see Supplementary Fig. 1). Specifically, this model allowed us to assess whether the strength or direction of the interaction between X and W (first moderator) varies as a function of Z (second moderator). The model evaluates three key interaction terms: X×W, X×Z and X×W×Z, the last of which is the focal term that indicates a moderated moderation effect. In this study, “Ammonia or EHC” was the independent variable (X), “SPH or SMH” was the dependent variable (Y), “Group: with or without MHE” was the first moderator (W) and “Sex” was the second moderator (Z). Standardized values were used to perform the analysis to facilitate interpretation. Bias-corrected bootstrapping with 5,000 iterations was employed to assess the significance of the moderating effects. Confidence intervals that did not include zero were interpreted as statistically significant.

Age and educational level were added as covariates, provided age was significantly correlated with the four EHC outcomes (aiming: *r*=-0.29, *p*<0.01; tapping: *r*=-0.38, *p*<0.01; bimanual coordination: *r*=0.30, *p*<0.01, visuomotor coordination *r*=0.32, *p*<0.01) and educational level presented between-group differences, with controls having higher levels than MHE and NMHE (F= 5.52, *p*=0.005). To perform these analyses, all data were transformed to Z scores.

**SUPPLEMENTARY FIGURES**

**
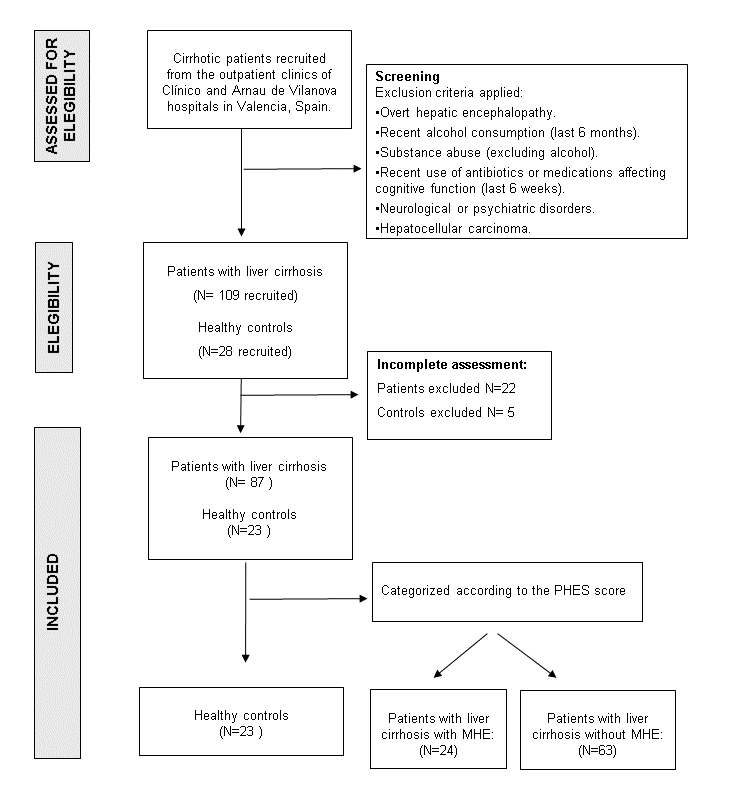
**

**Supplementary Figure 1.** Flow chart of the steps followed in the selection of the study sample


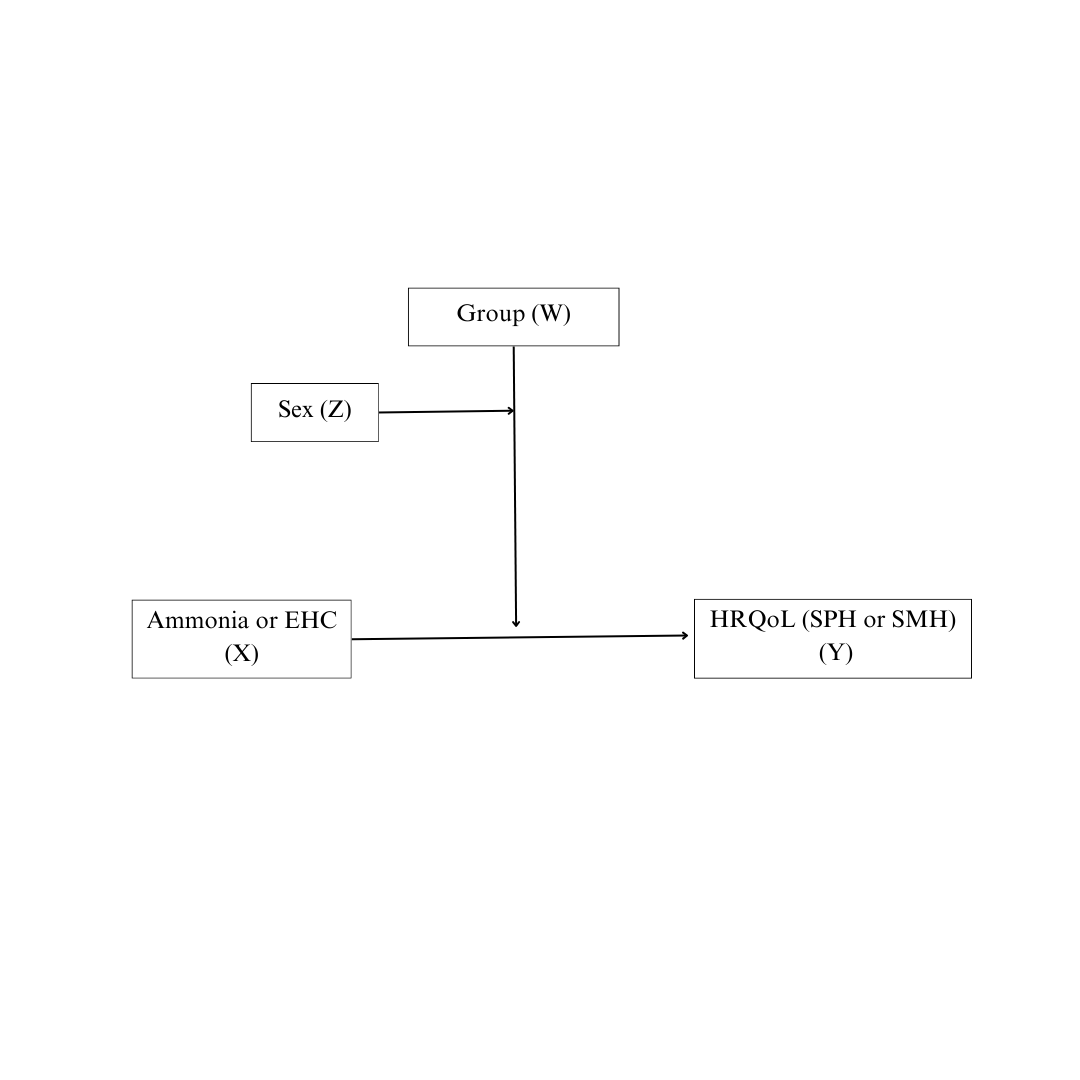


**Supplementary Figure 2.** Moderated moderation model. EHC, eye-hand coordination; HRQoL, health-related quality of life; SPH, subjective physical health; SPM, subjective mental health

**Supplementary Table 1.** Blood ammonia levels according to sex in the three study groups

|  | **Sex** | **Controls** | **NMHE patients** | **MHE patients** | ***Global ANOVA p*-value** |
| --- | --- | --- | --- | --- | --- |
| **Blood ammonia (µM)** | Women | 10.2 ± 1.5 | 23.6 ± 6.4 | 34.7 ± 11.9* | 0.075 |
|  | Men | 13.1 ± 2.3 | 29.3 ± 5 | 40.7 ± 6.2** | 0.064 |

Values are expressed as mean ± SEM. Abbreviations: NMHE and MHE, patients without and with minimal hepatic encephalopathy according to PHES score. Significant differences between patients and controls of the same sex are indicated with asterisks: *p<0.05; **p<0.01.

**Supplementary Table 2.** Statistics of the interaction of ammonia levels in moderated moderation analysis

| Ammonia levels | | | | |  | | |  |  |  | | | |  | |
| --- | --- | --- | --- | --- | --- | --- | --- | --- | --- | --- | --- | --- | --- | --- | --- |
| SPH |  | | ***B*** | **SE** | | | | **t** | | | ***p*** | | | ***CI 95%*** | |
|  | **Interactions** |  | | | |  |  | | | | |  |  | |  |
|  | Ammonia × Group | -148.596 | | | | 64.420 | -2.307 | | | | | **0.023** | -276.62, -20.57 | |  |
|  | Ammonia × Sex | -210.165 | | | | 99.785 | -2.106 | | | | | **0.038** | -408.47, -11.86 | |  |
|  | Group × Sex | -20.131 | | | | 58.841 | -0.034 | | | | | 0.733 | -137.07, 96.80 | |  |
|  | Ammonia × Group × Sex | 179.208 | | | | 72.972 | 2.456 | | | | | **0.016** | 34.19, 324.22 | |  |
| SMH | Ammonia × Group | -6.252 | | | | 2.091 | -2.991 | | | | | **0.004** | -10.41, -2.10 | |  |
|  | Ammonia × Sex | -8.066 | | | | 3.238 | -2.491 | | | | | **0.014** | -14.50, -1.63 | |  |
|  | Group × Sex | -179.014 | | | | 74.936 | -2.389 | | | | | **0.019** | -327.93, -30.09 | |  |
|  | Ammonia × Group × Sex | 6.709 | | | | 2.368 | 2.833 | | | | | **0.005** | 2.00, 11.42 | |  |

Abbreviations: SPH, subjective physical health; SMH, subjective mental health; SE, standard error. Significant *P* values (p<0.05) are in bold.

**Supplementary Table 3.** Statistics of the interaction of EHC indices in moderated moderation analysis

| AIMING | | | | |  | | |  |  |  | | | |  | |  |  |  |  |  |  |
| --- | --- | --- | --- | --- | --- | --- | --- | --- | --- | --- | --- | --- | --- | --- | --- | --- | --- | --- | --- | --- | --- |
| SPH |  | | ***B*** | **SE** | | | | **t** | | | ***p*** | | | ***CI 95%*** | |  |  |  |  |  |  |
|  | **Interactions** |  | | | |  |  | | | | |  |  | |  |  |  |  |  |  |  |
|  | AI × Group | 5.977 | | | | 4.907 | 1.218 | | | | | 0.227 | -3.776, 15.733 | |  |  |  |  |  |  |  |
|  | AI × Sex | 9.115 | | | | 7.577 | 1.203 | | | | | 0.232 | -5.948, 24.178 | |  |  |  |  |  |  |  |
|  | Group × Sex | 49.396 | | | | 87.967 | 0.562 | | | | | 0.576 | -125.478, 224.270 | |  |  |  |  |  |  |  |
|  | AI × Group × Sex | -10.611 | | | | 5.781 | -1.836 | | | | | 0.070 | -19.8142, 1.865 | |  |  |  |  |  |  |  |
|  | Test of conditional AI × Group interaction by Sex | | | | | | | | | | | | | |  |  |  |  |  |  |  |
|  | Men | -4.633 | | | |  |  | | | | | 0.149 |  | |  |  |  |  |  |  |  |
|  | Women | 5.977 | | | |  |  | | | | | 0.227 |  | |  |  |  |  |  |  |  |
| SMH | AI × Group | 6.943 | | | | 4.649 | 1.493 | | | | | 0.139 | -2.298, 16.185 | |  |  |  |  |  |  |  |
|  | AI × Sex | 9.111 | | | | 7.178 | 1.269 | | | | | 0.208 | -5.159, 23.380 | |  |  |  |  |  |  |  |
|  | Group × Sex | 63.849 | | | | 83.336 | 0.766 | | | | | 0.446 | -101.817, 229.516 | |  |  |  |  |  |  |  |
|  | AI × Group × Sex | -11.209 | | | | 5.477 | -2.047 | | | | | 0.044 | -22.096, -0.322 | |  |  |  |  |  |  |  |
|  | Test of conditional AI × Group interaction by Sex | | | | | | | | | | | | | |  |  |  |  |  |  |  |
|  | Men | -4.266 | | | |  |  | | | | | 0.160 |  | |  |  |  |  |  |  |  |
|  | Women | 6.943 | | | |  |  | | | | | 0.139 |  | |  |  |  |  |  |  |  |
| LINEAR TRACKING | | | | | | | | | | | | | | | |  |  |  |  |  |  |
| SPH | LTI × Group | **345.957** | | | | **138.863** | **2.491** | | | | | **0.015** | **70.038, 621.876** | |  |  |  |  |  |  |  |
|  | LTI × Sex | **703.383** | | | | **277.870** | **2.531** | | | | | **0.013** | **151.260, 1255.506** | |  |  |  |  |  |  |  |
|  | Group × Sex | -11.910 | | | | 65.447 | -0.182 | | | | | 0.856 | -141.952, 118.133 | |  |  |  |  |  |  |  |
|  | LTI × Group × Sex | **-337.184** | | | | **143.652** | **-2.347** | | | | | **0.021** | **-622.617, -51.750** | |  |  |  |  |  |  |  |
|  | Test of conditional LTI × Group interaction by Sex | | | | | | | | | | | | | |  |  |  |  |  |  |  |
|  | Men | 8.774 | | | |  |  | | | | | 0.812 |  | |  |  |  |  |  |  |  |
|  | Women | **345.957** | | | |  |  | | | | | **0.015** |  | |  |  |  |  |  |  |  |
| SMH | LTI × Group | **346.426** | | | | **133.317** | **2.599** | | | | | **0.011** | **81.528, 611.323** | |  |  |  |  |  |  |  |
|  | LTI × Sex | **688,335** | | | | **266.771** | **2.580** | | | | | **0.012** | **158.265, 1218.401** | |  |  |  |  |  |  |  |
|  | Group × Sex | 3.9200 | | | | 62.833 | 0.062 | | | | | 0.950 | -120.930,128.768 | |  |  |  |  |  |  |  |
|  | LTI × Group × Sex | **-326.731** | | | | **137.914** | **-2.369** | | | | | **0.020** | **-600.764,-52.699** | |  |  |  |  |  |  |  |
|  | Test of conditional LTI × Group interaction by Sex | | | | | | | | | | |  |  | |  |  |  |  |  |  |  |
|  | men | 19.694 | | | |  |  | | | | | 0.578 |  | |  |  |  |  |  |  |  |
|  | women | **346.426** | | | |  |  | | | | | **0.011** |  | |  | |  |  |  |  |  |
| STEADINESS | | | | | | | | | | | | | | | |  |  |  |  |  |  |
| SPH | SI × Group | **-10.691** | | | | **3.446** | **-3.102** | | | | | **0.003** | **-17.545,-3.837** | |  |  |  |  |  |  |  |
|  | SI × Sex | -10.428 | | | | 5.378 | -1.939 | | | | | 0.056 | -21.1237, 0.267 | |  |  |  |  |  |  |  |
|  | Group × Sex | **-366.173** | | | | **123.333** | **-2.969** | | | | | **0.004** | **-611.435, -120.911** | |  |  |  |  |  |  |  |
|  | SI × Group × Sex | **8.543** | | | | **3.8974** | **2.192** | | | | | **0.031** | **0.792, 16.293** | |  |  |  |  |  |  |  |
|  | Test of conditional SI × Group interaction by Sex | | | | | | | | | | |  |  | |  |  |  |  |  |  |  |
|  | men | -2.148 | | | |  |  | | | | | 0.228 |  | |  |  |  |  |  |  |  |
|  | women | **-10.691** | | | |  |  | | | | | **0.003** |  | |  |  |  |  |  |  |  |
| SMH | SI × Group | **-11.468** | | | | **3.219** | **-3.562** | | | | | **0.001** | **-17.871, -5.066** | |  |  |  |  |  |  |  |
|  | SI × Sex | **-11.449** | | | | **5.031** | **-2.276** | | | | | **0.025** | **-21.455,-1.44** | |  |  |  |  |  |  |  |
|  | Group × Sex | **-3984.787** | | | | **114.438** | **-3.449** | | | | | **0.001** | **-622.362, -167.214** | |  |  |  |  |  |  |  |
|  | SI × Group × Sex | **9.833** | | | | **3.641** | **2.700** | | | | | **0.008** | **2.593, 17.074** | |  |  |  |  |  |  |  |
|  | Test of conditional SI × Group interaction by Sex | | | | | |  | | | | |  |  | |  |  |  |  |  |  |  |
|  | Men | -1.635 | | | |  |  | | | | | 0.326 |  | |  |  |  |  |  |  |  |
|  | Women | **-11.468** | | | |  |  | | | | | **0.001** |  | |  |  |  |  |  |  |  |
| TAPPING | | | | | | | | | | | | | | | |  |  |  |  |  |  |
| SPH | TI × Group | **9.566** | | | | **2.946** | **3.247** | | | | | **0.002** | **3.707, 15.424** | |  |  |  |  |  |  |  |
|  | TI × Sex | **9.971** | | | | **3.702** | **2.693** | | | | | **0.008** | **2.609, 17.332** | |  |  |  |  |  |  |  |
|  | Group × Sex | **1222.667** | | | | **508.654** | **2.404** | | | | | **0.018** | **211.149, 2234.185** | |  |  |  |  |  |  |  |
|  | TI × Group × Sex | **-7.698** | | | | **3.089** | **-2.492** | | | | | **0.015** | **-13.841, -1.556** | |  |  |  |  |  |  |  |
|  | Test of conditional TI × Group interaction by Sex | | | | | |  | | | | |  |  | |  |  |  |  |  |  |  |
|  | Men | **1.867** | | | |  |  | | | | | **0.034** |  | |  |  |  |  |  |  |  |
|  | Women | **9.566** | | | |  |  | | | | | **0.002** |  | |  |  |  |  |  |  |  |
| SMH | TI × Group | **9.616** | | | | **2.683** | **3.584** | | | | | **0.001** | **1.284, 14.948** | |  |  |  |  |  |  |  |
|  | TI × Sex | **10.121** | | | | **3.392** | **2.984** | | | | | **0.004** | **3.379, 16.864** | |  |  |  |  |  |  |  |
|  | Group × Sex | **1266.066** | | | | **459.356** | **2.756** | | | | | **0.007** | **353.049, 2179.083** | |  |  |  |  |  |  |  |
|  | TI × Group × Sex | **-7.856** | | | | **2.801** | **-2.805** | | | | | **0.006** | **-13.423,-2.290** | |  |  |  |  |  |  |  |
|  | Test of conditional TI × Group interaction by Sex | | | | | |  | | | | |  |  | |  |  |  |  |  |  |  |
|  | Men | **1.759** | | | |  |  | | | | | **0.029** |  | |  |  |  |  |  |  |  |
|  | Women | **9.616** | | | |  |  | | | | | **0.001** |  | |  |  |  |  |  |  |  |
| VISUOMOTOR COORDINATION | | | | | | | | | | | | | | | |  |  |  |  |  |  |
| SPH | VC × Group | 4.337 | | | | 4.517 | 0.960 | | | | | 0.011 | -376.946, -48.045 | |  |  |  |  |  |  |  |
|  | VC × Sex | 7.834 | | | | 7.280 | 1.076 | | | | | 0.069 | -513.676, 20.157 | |  |  |  |  |  |  |  |
|  | Group × Sex | 40.821 | | | | 86.158 | 0.474 | | | | | 0.070 | -1023.522, 41.490 | |  |  |  |  |  |  |  |
|  | VC × Group × Sex | -8.975 | | | | 5.455 | -1.645 | | | | | 0.069 | -13.565, 341.594 | |  |  |  |  |  |  |  |
|  |  |  | | | |  |  | | | | |  |  | |  |  |  |  |  |  |  |
|  | Test of conditional VC × Group interaction by Sex | | | | | | | | | | | |  | |  |  |  |  |  |  |  |
|  | Men | -48.481 | | | |  |  | | | | | 0.169 |  | |  |  |  |  |  |  |  |
|  | **Women** | **-212.495** | | | |  |  | | | | | **0.012** |  | |  |  |  |  |  |  |  |
| SMH | VC × Group | **-229.949** | | | | **79.239** | **-2.902** | | | | | **0.005** | **-387.421, -72.476** | |  |  |  |  |  |  |  |
|  | VC × Sex | **-276.264** | | | | **128.612** | **-2.148** | | | | | **0.035** | **-531.855, -20.674** | |  |  |  |  |  |  |  |
|  | Group × Sex | **-580.045** | | | | **256.585** | **-2.261** | | | | | **0.026** | **-1089.956, -70.135** | |  |  |  |  |  |  |  |
|  | VC × Group × Sex | **195.936** | | | | **85.566** | **2.289** | | | | | **0.024** | **25.892, 365. 979** | |  |  |  |  |  |  |  |
|  | Test of conditional VC × Group interaction by Sex | | | | | |  | | | | |  |  | |  |  |  |  |  |  |  |
|  | Men | -34.013 | | | |  |  | | | | | 0.313 |  | |  |  |  |  |  |  |  |
|  | Women | **-229.948** | | | |  |  | | | | | **0.005** |  | |  |  |  |  |  |  |  |
| BIMANUAL COORDINATION | | | | | | | | | | | | | | | |  |  |  |  |  |  |
| SPH | BC × Group | **-505.892** | | | | **138.160** | **-3.662** | | | | | **0.004** | **-780.457, -231.327** | |  |  |  |  |  |  |  |
|  | BC × Sex | **-518.071** | | | | **202.520** | **-2.558** | | | | | **0.012** | **-920.537, -115.605** | |  |  |  |  |  |  |  |
|  | Group × Sex | **-1173.772** | | | | **371.392** | **-3.161** | | | | | **0.002** | **-1911.837, -435.707** | |  |  |  |  |  |  |  |
|  | BC × Group × Sex | **431.142** | | | | **143.735** | **2.999** | | | | | **0.004** | **145.499, 7.9156** | |  |  |  |  |  |  |  |
|  | Test of conditional BC × group interaction by sex | | | | | |  | | | | |  |  | |  |  |  |  |  |  |  |
|  | Men | -74.751 | | | |  |  | | | | | 0.063 |  | |  |  |  |  |  |  |  |
|  | Women | **-505.892** | | | |  |  | | | | | **0.004** |  | |  |  |  |  |  |  |  |
| SMH | BC × Group | **-448.144** | | | | **134.778** | **-3.325** | | | | | **0.001** | **-715.988, -180.299** | |  |  |  |  |  |  |  |
|  | BC × Sex | **-436.745** | | | | **197.562** | **-2.211** | | | | | **0.023** | **-829.359, -44.131** | |  |  |  |  |  |  |  |
|  | BC × Sex | **-1008.249** | | | | **362.300** | **-2.783** | | | | | **0.007** | **-1728.247,-288.253** | |  |  |  |  |  |  |  |
|  | BC × Group × Sex | **-371.922** | | | | **140.216** | **2.653** | | | | | **0.010** | **93.272, 650.573** | |  |  |  |  |  |  |  |
|  | Test of conditional BC × Group interaction by Sex | | | | | |  | | | | |  |  | |  |  |  |  |  |  |  |
|  | Men | -76.221 | | | |  |  | | | | | 0.052 |  | |  |  |  |  |  |  |  |
|  | Women | **-448.144** | | | |  |  | | | | | **0.001** |  | |  |  |  |  |  |  |  |

Note: SPH, subjective physical health; SMH, subjective mental health; AI, aiming index; LTI, linear tracking index; SI, steadiness index; TI, tapping index; VC, visuomotor coordination; BC, bimanual coordination; LLCI, lower limit confidence interval; ULCI, upper limit confidence interval. Significant interactions are in bold.

**Supplementary Table 4.** Complete sample blood ammonia levels, performance in eye-hand coordination tests, and SF-36 scores in the three study groups

| Measure | Controls (N=varies) | NMHE (N=varies) | MHE (N=varies) | F(df, RSE) | Global ANOVA p | Effect Size (NP2) |
| --- | --- | --- | --- | --- | --- | --- |
| Blood Ammonia (µM) | 11.48 ± 5.36 (N=26) | 27.11 ± 3.1* (N=76) | 42.16±5.17^***α^ (N=28) | 8.48 (2,126) | <0.001 | 0.12 |
| Vienna Test System | | | | | | |
| Aiming Index (nh/t) | 1.98±0.1 (N=23) | 1.66±0.1^**^ (N=63) | 1.18±0.1^*** ααα^ (N=24) | 20.61 (2,102) | 0.001 | 0.288 |
| Linear Tracking Index (ne/t) | 0.57±0.23 (N=23) | 0.34±0.13 (N=71) | 0.55±0.2 (N=24) | 0.48 (2,114) | 0.617 | 0.010 |
| Steadiness Index (ne) | 12.65±3.27 (N=23) | 18.79±1.87 (N=71) | 28.52±3.2^**^ (N=24) | 8.08 (2,102) | 0.003 | 0.098 |
| Tapping Index (nh) | 173.5 ± 7.45 (N=24) | 165.8 ± 4.42 (N=68) | 148.1±5.1^*^ (N=24) | 15.20 (2,102) | 0.027 | 0.063 |
| Coordination Tests | | | |  |  |  |
| Bimanual Coordination (min) | 2.03±0.13 (N=26) | 2.32±0.08 (N=75) | 3.3±0.13^*** ααα^ (N=27) | 28.76 (2,124) | <0.001 | 0.317 |
| Visuomotor Coordination (min) | 2.36±0.13 (N=24) | 2.80±0.08^**^ (N=68) | 4.01±0.13^***ααα^ (N=24) | 25.65 (2,107) | <0.001 | 0.415 |
| SF-36 Test | | | | | | |
| Subjective Physical Health | 254.1±39.0  (N=23) | 315.4±17.8  (N=63) | 252.2±28.9  (N=24) | 2.27(2,97) | 0.108 | 0.045 |
| Subjective Mental Health | 226.8±38.1  (N=23) | 335.6±17.3^*^  (N=63) | 282.5±28.1  (N=24) | 3.945(2,97) | 0.023 | 0.075 |

Values are expressed as mean ± SEM. Abbreviations: NMHE and MHE, patients without and with minimal hepatic encephalopathy according to PHES score, df: degrees of freedom, RSE: residual standard error, *n_p_^2^*: partial eta squared, t: time taken for the test, ne: number of errors, nh: number of hits, min: total minutes employed in the tests. Significant differences compared to controls are indicated by asterisks: **p* < 0.05; ***p* < 0.01; ****p* < 0.001; and from NMHE patients by α. (α: *p* < 0.05; ***/ααα: *p* < 0.001).

**STROBE Statement—Checklist of items that should be included in reports of *cross-sectional studies***

|  | Item No | Recommendation | Line / page |
| --- | --- | --- | --- |
| **Title and abstract** | 1 | (*a*) Indicate the study’s design with a commonly used term in the title or the abstract | 1-2 / 1 |
|  |  | (*b*) Provide in the abstract an informative and balanced summary of what was done and what was found | 34-58 / 2 |
| Introduction | | |  |
| Background/rationale | 2 | Explain the scientific background and rationale for the investigation being reported | 82-149 / 3-6 |
| Objectives | 3 | State specific objectives, including any prespecified hypotheses | 137-149/ 5-6 |
| Methods | | |  |
| Study design | 4 | Present key elements of study design early in the paper | 153-154 / 6 |
| Setting | 5 | Describe the setting, locations, and relevant dates, including periods of recruitment, exposure, follow-up, and data collection | 154-168 / 6 |
| Participants | 6 | (*a*) Give the eligibility criteria, and the sources and methods of selection of participants | 154-164 / 6 |
| Variables | 7 | Clearly define all outcomes, exposures, predictors, potential confounders, and effect modifiers. Give diagnostic criteria, if applicable | 176-216/ 7-8 |
| Data sources/ measurement | 8* | For each variable of interest, give sources of data and details of methods of assessment (measurement). Describe comparability of assessment methods if there is more than one group | 223-243/ 8-9 |
| Bias | 9 | Describe any efforts to address potential sources of bias | 248-250 / 9 |
| Study size | 10 | Explain how the study size was arrived at | 244-247 / 9 |
| Quantitative variables | 11 | Explain how quantitative variables were handled in the analyses. If applicable, describe which groupings were chosen and why | 223-243/ 8-9 |
| Statistical methods | 12 | (*a*) Describe all statistical methods, including those used to control for confounding | 223-250/ 8-9  Supplementary methods |
|  |  | (*b*) Describe any methods used to examine subgroups and interactions | 223-250/8-9  Supplementary methods |
|  |  | (*c*) Explain how missing data were addressed | 223-250/8-9 |
|  |  | (*d*) If applicable, describe analytical methods taking account of sampling strategy | 223-250/8-9 |
|  |  | (*e*) Describe any sensitivity analyses | 223-250/8-9 |
| Results | | |  |
| Participants | 13* | (a) Report numbers of individuals at each stage of study—eg numbers potentially eligible, examined for eligibility, confirmed eligible, included in the study, completing follow-up, and analysed | 217-221 / 8  Supplementary Fig. 1 |
|  |  | (b) Give reasons for non-participation at each stage | 217-221 / 8 |
|  |  | (c) Consider use of a flow diagram | Supplementary Fig. 1 |
| Descriptive data | 14* | (a) Give characteristics of study participants (eg demographic, clinical, social) and information on exposures and potential confounders | Table 1  (p. 27) |
|  |  | (b) Indicate number of participants with missing data for each variable of interest | 217-221 / 8 |
| Outcome data | 15* | Report numbers of outcome events or summary measures | 253-277/ 9-10 |
| Main results | 16 | (*a*) Give unadjusted estimates and, if applicable, confounder-adjusted estimates and their precision (eg, 95% confidence interval). Make clear which confounders were adjusted for and why they were included | 254-318/ 9-11  Tables 2-7 (pp. 28-34)  Supplementary Tables 1-4 |
|  |  | (*b*) Report category boundaries when continuous variables were categorized | - |
|  |  | (*c*) If relevant, consider translating estimates of relative risk into absolute risk for a meaningful time period | - |
| Other analyses | 17 | Report other analyses done—eg analyses of subgroups and interactions, and sensitivity analyses | Tables 2-7  (pp. 28-34)  Supplementary Tables 1-4 |
| Discussion | | |  |
| Key results | 18 | Summarise key results with reference to study objectives | 321-329/12 |
| Limitations | 19 | Discuss limitations of the study, taking into account sources of potential bias or imprecision. Discuss both direction and magnitude of any potential bias | 434-453/15-16 |
| Interpretation | 20 | Give a cautious overall interpretation of results considering objectives, limitations, multiplicity of analyses, results from similar studies, and other relevant evidence | 330-433/12-16 |
| Generalisability | 21 | Discuss the generalisability (external validity) of the study results | 458-474/16 |
| Other information | | |  |
| Funding | 22 | Give the source of funding and the role of the funders for the present study and, if applicable, for the original study on which the present article is based | 709-719/23 |

*Give information separately for exposed and unexposed groups.

**Note:** An Explanation and Elaboration article discusses each checklist item and gives methodological background and published examples of transparent reporting. The STROBE checklist is best used in conjunction with this article (freely available on the Web sites of PLoS Medicine at http://www.plosmedicine.org/, Annals of Internal Medicine at http://www.annals.org/, and Epidemiology at http://www.epidem.com/). Information on the STROBE Initiative is available at www.strobe-statement.org.
